# Characteristics of guided modes in graphene-coated chiral nihility fibers

**DOI:** 10.1371/journal.pone.0315449

**Published:** 2025-03-21

**Authors:** Muhammad Usman Shahid, Abdul Ghaffar, Majeed A. S. Alkanhal, Yasin Khan, Muhammad Amir Ali

**Affiliations:** 1 Department of Physics, University of Agriculture, Faisalabad, Pakistan; 2 Department of Electrical Engineering, King Saud University, Riyadh, Saudi Arabia; 3 School of Materials Science and Engineering, Zhengzhou University, Zhengzhou, China; University of Lucknow, INDIA

## Abstract

In this paper, guided modes in isotropic chiral nihility metamaterial fibers coated with graphene layers supported by conventional dielectric material are studied theoretically. Kubo formalism is applied to model the conductivity of single-layer graphene, and suitable boundary conditions are applied at the proposed waveguide structure. The dispersion equation for the chiral nihility fiber and the normalized cut-off frequency are derived. Characteristic curves for forward and backward modes, the effect of the graphene layer, the influence of the chirality parameter, and the energy flow distribution of several lower-order guided modes $\left(m=0,1,-1\right)$ in chiral nihility fibers are investigated briefly. It is found that the band gap between the curves of forward and backward modes may be tuned by radial distance, chiral strength, and chemical potential. By the addition of a graphene medium with chiral nihility, some novel properties of the chiral nihility fibers, such as anomalous dispersion and higher wave propagation, are examined. This graphene- and negative index material (NIM)-based structure makes it applicable for developing novel fiber devices, communication systems, and chiral sensing systems at the terahertz (THz) frequency.

## Introduction

Negative index materials (NIMs) (also called left-handed materials) have gained much importance during the last decade due to their novel properties, such as their perfect lens [1–3], optical clocking capabilities [4, 5] and enhance sensitivity to material parameters [6–8]. Various types of waveguides having NIMs have been discussed in the literature [9–15]. It has been found that NIMs have many extraordinary and mind-blowing potential applications in the electromagnetic (EM) field, such as slow light devices, perfect lens systems, and sensor systems. Moreover, NIMs in slab and circular waveguides have become an interesting related topic. Many researchers have intensively used NIMs in the core and cladding of waveguides to investigate the different wave applications. In guided and surface modes, many unusual characteristics have been examined while using NIM waveguides [15–17]. For example, the EM waves in such waveguides are very slow and can even stop [18, 19]. At first, the study of NIMs was only theoretical, but later, in 2009, these materials were developed in chiral metamaterials experimentally at the microwave [20] and terahertz (THz) frequencies [21]. The negative indexes of circularly polarized waves in chiral metamaterials are due to the large chirality parameter.

Lakhtakia [22] introduced the new “nihility” material having zero permittivity and permeability simultaneously. Then, Tretyakov et al. developed the chiral nihility material and introduced the chiral nihility concept [23]. A chiral nihility material is a material having properties ε=0, μ=0, and κ≠0, where *κ* is the chirality parameter at a certain frequency [23–25]. For this reason, this material does not permit EM waves to pass through it. Therefore, Maxwell equations can also be reduced under the nihility conditions as follows:


∇×E =0

∇×H =0.

It has been observed that no propagation is possible in a nihility medium because of the zero value of the wave number corresponding to the medium [26]. They conclude that the unbonded chiral nihility medium support two modes of circular polarization; one forward and other backward. Although the field of chiral nihility has not yet been realized, chiral nihility has attracted the attention of many researchers [27–31]. The chiral nihility is still a developing material and the chiral nihility has a great requirement that the permittivity and permeability are very small. In this regard, gyrotropic chiral (G-chiral) and non-reciprocating chiral materials are proposed for optical applications [3,32]. Waves and their energies are driven into a chiral nihility, indicating that the eigenwaves are still circularly polarized, but one is a backward wave, whose velocity phase is anti-parallel to the corresponding Poynting vector. Another interesting phenomenon is the negative reflection of the two eigenwaves in the medium between chiral nihility and perfect electric conductor, which has still never been observed in ordinary materials [33].

Chiral nihility-filled waveguides have drawn the attention of researchers in the optoelectronic community due to their performance in sensors [34–36]. The novel features of chiral nihility-based waveguides for guided modes have been theoretically examined [37, 38]. The conditions for chiral slab waveguides and surface modes of chiral media for surface plasmon polaritons have been studied [37]. By using chiral negative refraction, Dong examined the characteristics of guided modes for parallel plate, circular, slab, and grounded slab waveguides [39–41]. A strong chiral core in a planner dielectric waveguide can support a single-mode backward guided wave [38]. A parallel plate waveguide structure with a grounded chiral nihility slab [39] and a two-layer chiral nihility material with one layer of air [42] have also been studied. These types of waveguide structures can be applied to microwave and millimeter integrated circuits [42]. In [43], 3D negative refractive index isotropic chiral metamaterials were investigated.

Chiral nihility characterized by ε=0, μ=0, serves as a transition phase to NIMs as *ε* and *μ* approaches zero. The refractive index (*n*) transitions from positive to negative, governed by the equation $n=\sqrt{\varepsilon \mu }$, when *ε* and *μ* are precisely zero n=0, marking the chiral nihility point introducing small negative values of *ε* and *μ* yields n<0, characteristic of NIMs. This correlation is mathematically expressed as:


$$n=\sqrt{\varepsilon \mu }$$

When n≈0 (chiral nihility)

When n<0 (NIMs, for ε<0 and μ<0)

Graphene is a form of 2D allotropic carbon having a single-atom thickness of graphite packed in a sp^2^ atomic bond with a hexagonal pattern. Since 2004 when graphene was first isolated, its research has expanded very quickly. Surprisingly, high-quality graphene makes the research easy due to the theoretical description of its composition, properties, and structure [44–46]. The most valuable property, which we mostly adopt in our research, is that it is a zero-overlap semimetal (for both types of charge carriers, electrons and holes) with high electrical conductivity [47]. The plasmonic material-like properties of this multipurpose nanomaterial in the THz and infrared spectrum range make it more common and more general. With these attractive and supportive surface wave properties, like low losses and high field confinement, graphene opens new doors for ultra-high speeds, low energy consumption, and small wave dimensions in terahertz and infrared plasmonic applications [48]. These unusual properties have made graphene a subject of study in electronics, and Konstantin Novoselov and Andre Geimin were awarded the Nobel Prize in Physics in 2010 for experiments based on graphene [49].

Although graphene is as thin as a carbon atom, two hundred times stronger than steel and has greater electrical conductivity than other materials at room temperature. Due to the simple and ordered structure, due to the closeness of carbon atom, this material has better characteristics, such as tunability, high sensitivity of biomolecule absorption, low ohmic loss, and high flexibility [50].

A single layer of graphene behaves like a semiconductor with a low chemical potential, but by applying a voltage or adding impurities it behaves like a conductor and can support surface plasmon polarons. This change in graphene’s behavior from a semiconductor to a conductor is caused by a change in its Fermi energy [51]. According to studies on graphene’s electronic sensitivity to biomolecule adsorption, graphene absorbs biomolecules much more strongly than metal surfaces [52]. Therefore, due to good surface adhesion and other specific properties of graphene compared to gold it is a very biocompatible material [53]. It is expected that the adsorption efficiency of graphene surface will be higher than that of gold, and in this case the sensitivity of the graphene-based biosensor will be higher than the reported gold biosensors [51,54,55].

This paper discusses the EM wave propagation and energy flux from an infinite uniaxial chiral nihility metamaterial-filled cylinder having a circular cross-section that is uniformly coated with graphene layers. The geometry of this problem is divided into three parts: chiral nihility core, graphene coating layer, and free space. Suitable boundary conditions are used at the cylindrical interface. The numerical work is compared with the literature for some cases to verify the numerical codes and analytical formulations. Throughout the article, the $\text{exp}\left(j\omega t\right)$ time dependence is suppressed for simplification.

## Formulations

**Fig 1:**
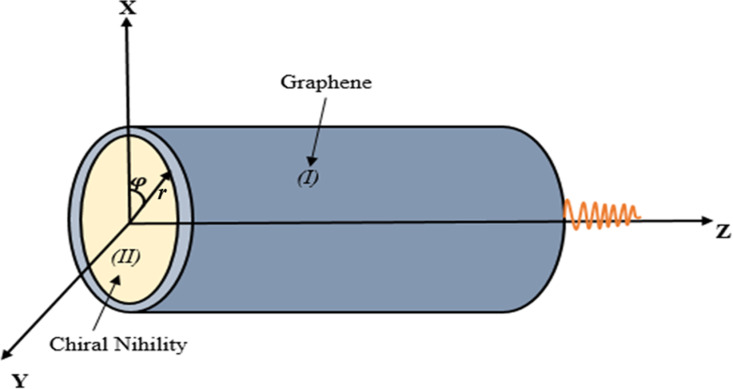
Waveguide Geometry. Cylindrical waveguide coated with graphene and filled with chiral nihility metamaterial.

Consider the graphene-coated chiral nihility material-filled waveguide shown in Fig 1. Material (I) in the cladding is the conductive layer of graphene, which is supported by a conventional dielectric material having $\varepsilon_{I}$, $\mu_{I}$ permittivity and permeability. Material (II) on the core is the isotropic chiral nihility metamaterial whose permittivity and permeability are simultaneously zero but chirality parameter is non-zero as $\varepsilon_{II}=0$, $\mu_{II}=0$, and κ≠0 at a specific frequency [23]. “r” is the radius of the cylinder, and the cladding is assumed to extend infinitely.

For the isotropic chiral nihility metamaterial, we select the appropriate constitutive relations, which are given as [23]


$$D=\varepsilon E-j\kappa \sqrt{\varepsilon_{0}\mu_{0}}H,B=\mu H+j\kappa \sqrt{\varepsilon_{0}\mu_{0}}E,$$ (1)

where “$\varepsilon_{0}$” and “$\mu_{0}$” denote the permittivity and permeability of free space and “*κ*” is the chirality admittance.

The graphene is molded in a highly conductive thin layer of negligible thickness having conductivity “$\sigma_{g}$” according to Kubo formalism as [56]


$$\sigma_{g}=j\frac{TK_{B}e^{2}}{\pi \hbar^{2}\left(\omega +\frac{j}{{\text{Γ}}_{1}}\right)}\left(\frac{\mu_{c}}{K_{B}T}+2\text{Log}\left[e^{-\frac{\mu_{c}}{K_{B}T}}+1\right]\right)+j\frac{e^{2}}{4\text{Pi}\hbar }\text{Log}\left[\frac{2\left[\mu_{c}\right]-\hbar \left(\omega +\frac{j}{{\text{Γ}}_{2}}\right)}{2\left[\mu_{c}\right]+\hbar \left(\omega +\frac{\text{i}}{{\text{Γ}}_{2}}\right)}\right],$$ (2)

where “$\mu_{c}$” is the chemical potential, “*T*” is the temperature, “$K_{B}$” is the Boltzmann constant, “*e*” is the charge on the electron, “*ℏ*” is the reduced Planck’s constant, and “${\text{Γ}}_{1,2}$” is the momentum relaxation time.

The electric and magnetic field components of the chiral nihility metamaterial can be separated [57] as shown:


$$E=E_{+}+E_{-},H=\frac{1}{\eta }\left(E_{+}+E_{-}\right),$$ (3)

where “*η*” denotes the wave impedance of the chiral nihility metamaterial, which is η=limμ→0,ε→0με. Thus, $E_{\pm }$ can satisfy the following equation:


$$\nabla \times E_{\pm }=\pm k_{\pm }E_{\pm }.$$ (4)

The transverse $E_{\pm t}$ and longitudinal $E_{\pm z}$ components of $E_{\pm }$ are separated as follows:


$$E_{\pm }=E_{\pm t}+\hat{z}E_{z}.$$ (5)

The relationship between transverse $E_{\pm t}$ and longitudinal $E_{\pm z}$ can also be derived from the following equation:


$$E_{\pm t}=\frac{1}{\kappa^{2}k_{0}^{2}-\beta^{2}}\left[-j\beta \nabla_{t}E_{\pm z}-\kappa k_{0}\hat{z}\times \nabla_{t}E_{\pm z}\right],$$ (6)

where $\nabla_{t}=\nabla -\hat{z}\frac{\partial }{\partial z}$, dependence on *z* is expressed as $\text{exp}\left(-j\beta z\right)$ and omitted for simplification, and *β* denotes the propagation constant. Here, we take the cylindrical coordinates $\left(\rho ,\varphi ,z\right)$, and the transverse EM components are written as follows:


$$\left(\begin{array}{c} E_{r}\\ H_{r}\\ E_{\varphi }\\ H_{\varphi } \end{array}\right)=\left(\begin{array}{c} \frac{-j\beta }{k_{r+}^{2}}\\ \frac{\beta }{\eta k_{r+}^{2}}\\ \frac{-k_{+}}{k_{r+}^{2}}\\ \frac{-jk_{+}}{\eta k_{r+}^{2}} \end{array}\begin{array}{c} \frac{k_{+}}{k_{r+}^{2}}\\ \frac{jk_{+}}{\eta k_{r+}^{2}}\\ \frac{-j\beta }{k_{r+}^{2}}\\ \frac{\beta }{\eta k_{r+}^{2}} \end{array}\begin{array}{c} \frac{-j\beta }{k_{r-}^{2}}\\ \frac{-\beta }{\eta k_{r-}^{2}}\\ \frac{k_{-}}{k_{r-}^{2}}\\ \frac{-jk_{-}}{\eta k_{r-}^{2}} \end{array}\begin{array}{c} \frac{-k_{-}}{k_{r-}^{2}}\\ \frac{jk_{-}}{\eta k_{r-}^{2}}\\ \frac{-j\beta }{k_{r-}^{2}}\\ \frac{-\beta }{\eta k_{r-}^{2}} \end{array}\right)\left(\begin{array}{c} \frac{\partial }{\partial r}E_{+z}\\ \frac{\partial }{r\partial \varphi }E_{+z}\\ \frac{\partial }{\partial r}E_{-z}\\ \frac{\partial }{r\partial \varphi }E_{-z} \end{array}\right).$$ (7)

From Equation (7), the wave equation in terms of $E_{\pm }$ can be obtained as follows:


$$(\nabla^{2}+k_{\pm }^{2})E_{\pm }=0.$$ (8)

For Region (II), the chiral nihility metamaterial, the longitudinal field components $E_{\pm z}$ are expressed as follows:


$$E_{+\text{z}\left(\text{I}\right)}=A_{1}J_{m}\left(k_{r+}r\right)\text{exp}\left(jm\varphi \right)$$ (9)

$$E_{-\text{z}\left(\text{I}\right)}=B{}_{1}J_{m}\left(k_{r-}r\right)\text{exp}\left(jm\varphi \right).$$ (10)

In the above equations, $A_{1}$, $B_{1}$ are the unknown constants, $J_{m}\left(\_\right)$ is the Bessel function of the first kind, *m* is the azimuthal field dependence (which may be a positive or negative integer), and $k_{r\mp }=\sqrt{k_{\mp }^{2}-\beta^{2}}=\sqrt{\kappa^{2}-{\left(\frac{\beta }{k_{0}}\right)}^{2}}=k_{r}$ are transverse wavenumbers of right circularly polarized (RCP) and left circularly polarized (LCP) waves in the core region. $k_{r}$ is a real number for guided mode (when $\beta <\kappa k_{0}$) and an imaginary number for surface mode (when $\beta >\kappa k_{0}$).

The EM field components for the chiral nihility metamaterial can be obtained from Equations (3) and (7).


$$E_{z}^{I}=\left(A_{1}+B_{1}\right)J_{m}\left(k_{r}r\right)\text{exp}\left(jm\varphi \right)$$ (11)

$$H_{z}^{I}=\frac{j}{\eta_{1}}\left(A_{1}-B_{1}\right)J_{m}\left(k_{r}r\right)\text{exp}\left(jm\varphi \right)$$ (12)

$$E_{r}^{I}=\left(A_{1}+B_{1}\right)\left(\frac{jm\kappa k_{0}}{k_{r}^{2}r}j_{m}\left(k_{r}r\right)-\frac{j\beta }{k_{r}}{j^{\prime }}_{m}^{}\left(k_{r}r\right)\right)\text{exp}\left(jm\varphi \right)$$ (13)

$$H_{r}^{I}=\frac{j}{\eta_{1}}\left(A_{1}-B_{1}\right)\left(\frac{jm\kappa k_{0}}{k_{r}^{2}r}j_{m}\left(k_{r}r\right)-\frac{j\beta }{k_{r}}{j^{\prime }}_{m}^{}\left(k_{r}r\right)\right)\text{exp}\left(jm\varphi \right)$$ (14)

$$E_{\phi }^{I}=\left(A_{1}+B_{1}\right)\left(\frac{\beta m}{k_{r}^{2}r}j_{m}\left(k_{r}r\right)-\frac{\kappa k_{0}}{k_{r}}{j^{\prime }}_{m}^{}\left(k_{r}r\right)\right)\text{exp}\left(jm\varphi \right)$$ (15)

$$H_{\phi }^{I}=\frac{j}{\eta_{1}}\left(A-B\right)\left(\frac{\beta m}{k_{r}^{2}r}j_{m}\left(k_{r}r\right)-\frac{\kappa k_{0}}{k_{r}}{j^{\prime }}_{m}^{}\left(k_{r}r\right)\right)\text{exp}\left(jm\varphi \right),$$ (16)

where $\eta_{1}$ is the wave impedance for the core material and ${j^{\prime }}_{m}^{}\left(\_\right)$ is the differentiation with respect to the argument of the Bessel function.

For the cladding material $\left(II\right)$, the EM field components are given follows:


$$E_{z}^{\text{II}}=\left(C_{1}+D_{1}\right)K_{m}\left(\tau_{2}r\right)\text{exp}\left(jm\varphi \right)$$ (17)

$$H_{z}^{\text{II}}=\frac{j}{\eta_{2}}\left(C_{1}-D_{1}\right)K_{m}\left(\tau_{2}r\right)\text{exp}\left(jm\varphi \right)$$ (18)

$$E_{r}^{II}=\left(\left(C_{1}-D_{1}\right)\frac{-jmk_{2}}{\tau_{2}^{2}r}K_{m}\left(\tau_{2}r\right)+\left(C_{1}+D_{1}\right)\frac{j\beta }{\tau_{2}}K_{m}^{'}\left(\tau_{2}r\right)\right)\text{exp }\left(jm\varphi \right)$$ (19)

$$H_{r}^{II}=\frac{j}{\eta_{2}}\left(\left(C_{1}+D_{1}\right)\frac{-jmk_{2}}{\tau_{2}^{2}r}K_{m}\left(\tau_{2}r\right)+\left(C_{1}-D_{1}\right)\frac{j\beta }{\tau_{2}}K_{m}^{'}\left(\tau_{2}r\right)\right)\text{exp}\left(jm\varphi \right)$$ (20)

$$E_{\phi }^{\text{II}}=\left(\left(C_{1}+D_{1}\right)\frac{-m\beta }{\tau_{2}^{2}r}K_{m}\left(\tau_{2}r\right)+\left(C_{1}-D_{1}\right)\frac{k_{2}}{\tau_{2}}K_{m}^{'}\left(\tau_{2}r\right)\right)\exp\left(jm\varphi \right)$$ (21)

$$H_{\phi }^{\text{II}}=\frac{j}{\eta_{2}}\left(\left(C_{1}-D_{1}\right)\frac{-m\beta }{\tau_{2}^{2}r}K_{m}\left(\tau_{2}r\right)+\left(C_{1}+D_{1}\right)\frac{k_{2}}{\tau_{2}}K_{m}^{'}\left(\tau_{2}r\right)\right)\text{exp}\left(jm\varphi \right),$$ (22)

where $C_{1},D_{1}$ are the unknown constants, $K_{m}\left(\_\right)$ and $K_{m}^{'}\left(\_\right)$ are the modified Bessel function of first kind and its derivative with respect to the argument, the transverse attenuation factor $\tau_{2}=k_{0}\sqrt{{\left(\frac{\beta }{k_{0}}\right)}^{2}-n_{2}^{2}}$ for the cladding material, $k_{2}$ is the wave number and $\eta_{2}$ is the admittance, and $n_{2}$ is the refractive index for the cladding.

To check the continuity of the tangential components $\left(E_{z},E_{\varphi },H_{z},H_{\varphi }\right)$, suitable boundary conditions are applied at interface r=a. From the solutions of Equations (11), (12), (17), and (18), we have four unknown constants: $A_{1}$, $B_{1}$, $C_{1}$, and $D_{1}$.


$$E_{z}^{I}=E_{z}^{\text{II}}$$ (23)

$$E_{\phi }^{I}=E_{\phi }^{\text{II}}$$ (24)

$$H_{z}^{I}-H_{z}^{\text{II}}=-\sigma_{T}E_{\phi }^{I}$$ (25)

$$H_{\phi }^{I}-H_{\phi }^{\text{II}}=\sigma_{T}E_{z}^{I},$$ (26)

where $\sigma_{T}=N\sigma_{g}$ and “N” indicates the number of graphene layers. From these constants, we obtain a homogeneous algebraic equation for the guided modes, which is expressed as follows:


$$\frac{m\beta }{a}\left(\frac{1}{\tau_{2}^{2}}+\frac{1}{k_{r}^{2}}\right)-\frac{k_{0}\kappa }{k_{r}}\frac{J_{m}^{'}\left(k_{r}a\right)}{J_{m}\left(k_{r}a\right)}=\pm \sqrt{\left(-\frac{k_{2}}{\tau_{2}}\frac{\eta_{2}\sigma_{g}}{a^{2}k_{r}^{4}}\frac{{K^{\prime }}_{m}^{}\left(\tau_{2}a\right)}{K_{m}\left(\tau_{2}a\right)}\left(\left(a^{2}k_{r}^{4}+m^{2}\beta^{2}\right)-2ak_{0}m\beta \kappa \frac{{J^{\prime }}_{m}^{}\left(k_{r}a\right)}{J_{m}\left(k_{r}a\right)}+a^{2}k_{0}^{2}k_{1}^{2}\kappa^{2}\frac{{J^{\prime }}_{m}^{2}\left(k_{r}a\right)}{J_{m}^{2}\left(k_{r}a\right)}\right)+\frac{k_{2}^{2}}{\tau_{2}^{2}}\frac{{K^{\prime }}_{m}^{2}\left(\tau_{2}a\right)}{K_{m}^{2}\left(\tau_{2}a\right)}\right)}.$$ (27)

The energy flux in the waveguide along the z-axis is expressed as follows:


$$S_{z}=\frac{1}{2}Re\left(E\times H^{*}\right)\cdot \hat{z}=\frac{1}{2}Re\left(E_{r}\times H_{\phi }^{*}-E_{\phi }\times H_{r}^{*}\right).$$ (28)

The energy flux for the core and cladding can be calculated from Equations (29) and (30):


$$S_{z1}=\frac{A_{1}^{2}+B_{1}^{2}}{4\eta_{1}k_{r}}\left({\left(k_{0}\kappa +\beta \right)}^{2}J_{m+1}^{2}\left(k_{r}r\right)-{\left(k_{0}\kappa -\beta \right)}^{2}J_{m-1}^{2}\left(k_{r}r\right)\right)$$ (29)

$$S_{z2}=\frac{C_{1}^{2}}{4\eta_{2}\tau_{2}^{2}}\left({\left(\beta +k_{2}\right)}^{2}K_{m+1}^{2}\left(\tau_{2}r\right)-{\left(\beta -k_{2}\right)}^{2}K_{m-1}^{2}\left(\tau_{2}r\right)\right)-\frac{D_{1}^{2}}{4\eta_{2}\tau_{2}^{2}}\left({\left(\beta -k_{2}\right)}^{2}K_{m+1}^{2}\left(\tau_{2}r\right)-{\left(\beta +k_{2}\right)}^{2}K_{m-1}^{2}\left(\tau_{2}r\right)\right).$$ (30)

## Results and discussion

**Fig 2:**
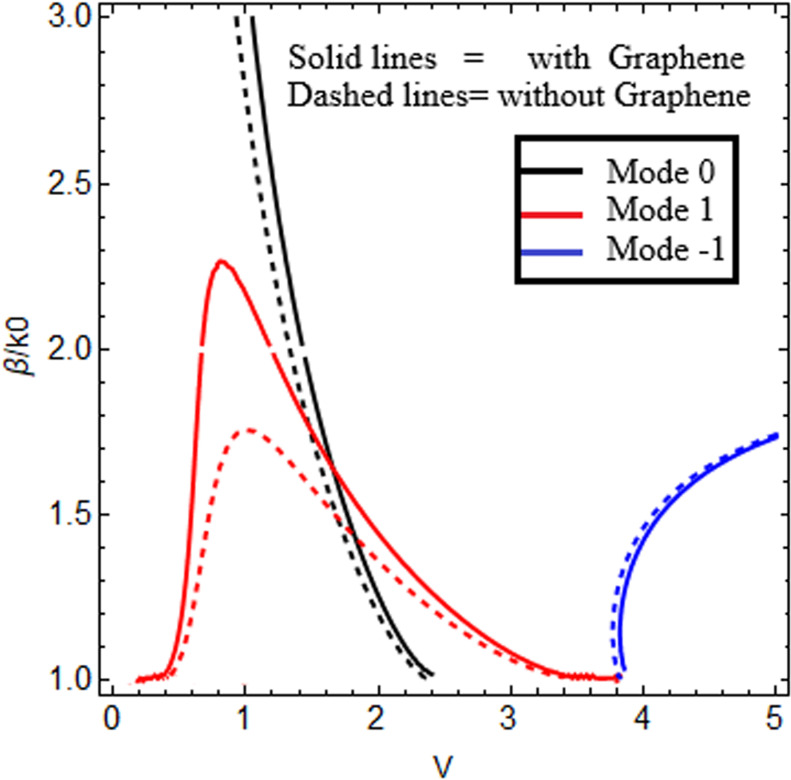
Comparison with literature. Propagation with and without graphene in cladding with chiral nihility metamaterial in core for lower-order modes (0, + 1, −1).

In this research work, we use graphene material ($\varepsilon_{I}$, $\mu_{I}$) as the cladding and chiral nihility metamaterial as the core ($\varepsilon_{II}=0$, $\mu_{II}=0$, κ≠0). The wave impedance of the chiral nihility metamaterial is $\eta_{II}=\eta_{0}$. Here, we discuss the effect of chirality by taking the following two chirality parameter values: κ=1.75,2.0. Other important factors used in this research include temperature *T = *273*K* and relaxation time $\Gamma_{1,2}=1ps$*.* We know that an EM wave can propagate through the chiral nihility metamaterial but it can only pass through in the form of two wave fields (forward field/mode and backward field/mode). We solve the dispersion equation (27) by taking both signs (positive and negative) of the right side of the equation for both forward and backward modes. The positive sign on the right side of the equation is used for forward mode, and the negative sign is used for backward mode. In Fig 2, we compare the effect of wave propagation for guided modes with and without graphene in the cladding. The solid curves show the influence of graphene (present work), and the dashed curves indicate the effect without graphene (published work). It is clearly observed from Fig 2 that the proposed waveguide structure (in Fig 1) can increase the wave propagation of EM waves. Graphene is remarkably effective at increasing wave propagation, which is the main objective of our research work. This comparison with published work confirms the accuracy and authenticity of the numerical results generated in the present work and ensures that this presented structure supports the hybrid guided modes.

**Fig 3:**
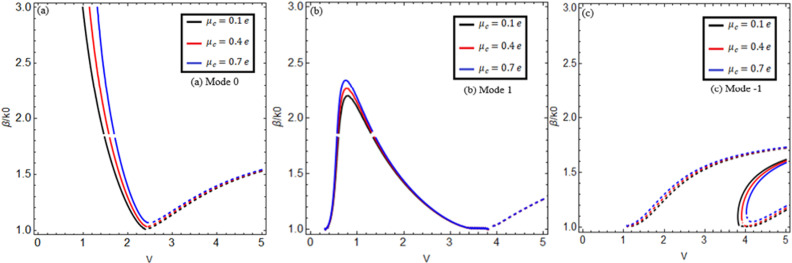
Effect of chemical potential. Change of chemical potential $\left(\mu_{c}=0.1e,0.4e,0.7e\right)$ between propagation constant versus normalized frequency $V(V=k_{0}a\sqrt{\kappa^{2}-n_{2}^{2}}$) for $\left(m=0,1,-1\right)$ at κ=1.75, *T = *273*K.* $\Gamma_{1,2}=1ps$.

**Fig 4:**
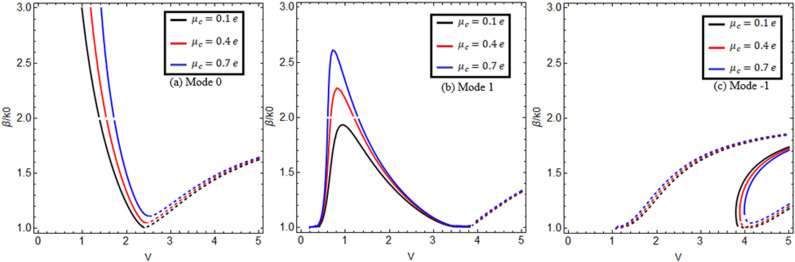
Effect of chemical potential. Change of chemical potential $\left(\mu_{c}=0.1e,0.4e,0.7e\right)$ between propagation constant versus normalized frequency $V(V=k_{0}a\sqrt{\kappa^{2}-n_{2}^{2}}$) for $\left(m=0,1,-1\right)$ at κ=2.0, *T = *273*K.* $\Gamma_{1,2}=1ps$.

Figs 3 and 4 reveal the effect of graphene’s chemical potential for two different chirality parameter values κ=1.75,2.0 between the normalized propagation constant $\left(\beta /k_{0}\right)$ versus normalized frequency $V(V=k_{0}a\sqrt{\kappa^{2}-n_{2}^{2}}$) for lower-order modes $\left(m=0,1,-1\right)$ in (a), (b), and (c). Here, we use normalized frequency in place of frequency because the chiral nihility metamaterial property occurs only at a certain frequency value. The solid black, red, and blue curves show the forward mode, and the dashed black, red, and blue curves indicate the backward mode of EM waves. Figs 3 and 4 show that increasing the chemical potential $\left(\mu_{c}=0.1e,0.4e,0.7e\right)$ increases the propagation constant $\beta /k_{0}$.

It is observed that for chirality parameter κ=1.75 in Fig 3(a) for mode m=0, the black, red, and blue curves explain the chemical potential behavior at $\mu_{c}=0.1e,0.4e,0.7e$. It is noted that the surface mode effects that exist below the normalized frequency at 1.32, 1.52, and 1.72 connect to the guided modes at critical points 1.32, 1.52, and 1.72. These critical points are the cross points of the surface mode and guided mode. This Fig shows that for forward mode, the dispersion curves have a negative slope when $\beta /k_{0}$ increases and then *V* starts decreasing from the normalized cut-off frequency $V_{c}$ = 2.417, and for backward mode, the dispersion curves have a positive slope when $\beta /k_{0}$ increases and then *V* starts increasing from the normalized cut-off frequency $V_{c}$ = 2.417, which is the first root of the Bessel function $J_{0}\left(V_{c}\right)=0$. In Fig 3(b) for mode m=1, the black, red, and blue curves explain the chemical potential behavior at $\mu_{c}=0.1e,0.4e,0.7e$. It is found that this mode exists between the $V_{c}$ = 0 and 3.82 normalized cut-off frequencies, which are the first and second roots of the Bessel function $J_{1}\left(V_{c}\right)=0$, and the surface mode effects that exist below the normalized cut-off frequencies at 0.21 and 1.2 connect to the guided modes at two critical points at 0.21 and 1.2. For this mode, it is evaluated that for forward mode, the dispersion curves first have a positive slope at $V_{c}$ = 0 when $\beta /k_{0}$ increases and then a negative slope at $V_{c}$ = 3.82 when $\beta /k_{0}$ decreases with the increase of *V*, and for backward mode, the dispersion curves have a positive slope when $\beta /k_{0}$ increases and then *V* also increases from the normalized cut-off frequency $V_{c}$ = 3.82. In Fig 3(c), for mode m=−1, the dispersion curves for forward mode have a positive slope when $\beta /k_{0}$ increases and then *V* starts increasing from the normalized cut-off frequency $V_{c}$ = 3.82, and for backward mode, the dispersion curves have a positive slope when $\beta /k_{0}$ increases and then *V* starts increasing from the normalized cut-off frequencies at $V_{c}$ = 1, 3.82, which is the second root of the Bessel function $J_{1}\left(V_{c}\right)=0$. When we select the chirality parameter κ=2.0 in Fig 4, we get the same results with a slightly higher band gap between the dispersion curves for all modes $\left(m=0,1,-1\right)$.

**Fig 5:**
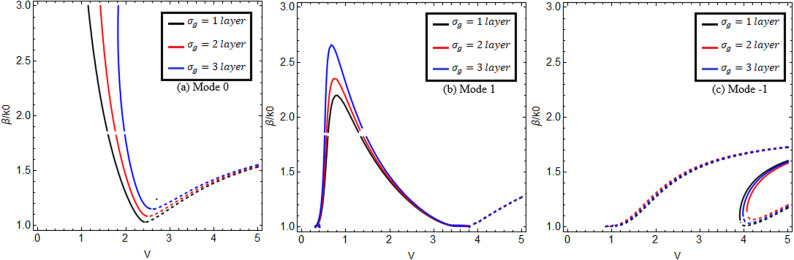
Effect of chemical potential. Change of chemical potential $\left(\mu_{c}=0.1e,0.4e,0.7e\right)$ between propagation constant versus normalized frequency $V(V=k_{0}a\sqrt{\kappa^{2}-n_{2}^{2}}$) for $\left(m=0,1,-1\right)$ at κ=1.75, *T = *273*K.* $\Gamma_{1,2}=1ps$.

**Fig 6:**
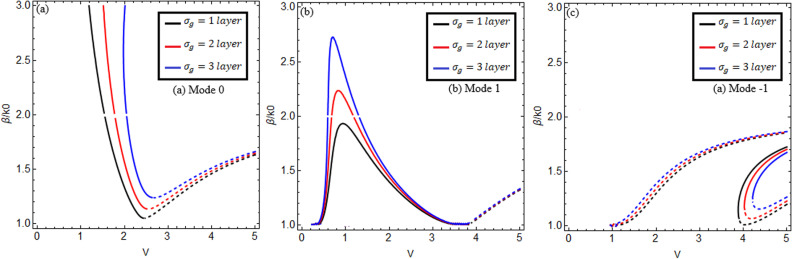
Effect of chemical potential. Change of chemical potential $\left(\mu_{c}=0.1e,0.4e,0.7e\right)$ between propagation constant versus normalized frequency $V(V=k_{0}a\sqrt{\kappa^{2}-n_{2}^{2}}$) for $\left(m=0,1,-1\right)$ at κ=2.0, *T = *273*K.* $\Gamma_{1,2}=1ps$.

Figs 5 and 6 investigate the effect of graphene layers in the cladding for chirality parameters κ=1.75,2.0, respectively, between the normalized propagation constant $\beta /k_{0}$ and versus normalized frequency $(V=k_{0}a\sqrt{\kappa^{2}-n_{2}^{2}}$) for lower-order modes $\left(m=0,1,-1\right)$ in (a), (b), and (c). The solid black, red, and blue curves explain the forward mode, and the dashed black, red, and blue curves indicate the backward mode of EM waves. Figs 5 and 6 show that in the case of forward mode, by increasing the number of graphene layers $\left(N=1,2,3\right)$, the propagation constant $\beta /k_{0}$ increases but moves toward the lower normalized frequency V, and in the case of backward mode, by increasing the number of graphene layers, the propagation constant $\beta /k_{0}$ increases while increasing the normalized frequency V. It can be observed from Figs 5 and 6 that increasing the chirality parameter from 1.75 to 2.0 increases the band gap between the dispersion curves. Both surface and guide modes are present in this case in $\left(m=0,1\right)$ modes.

**Fig 7:**
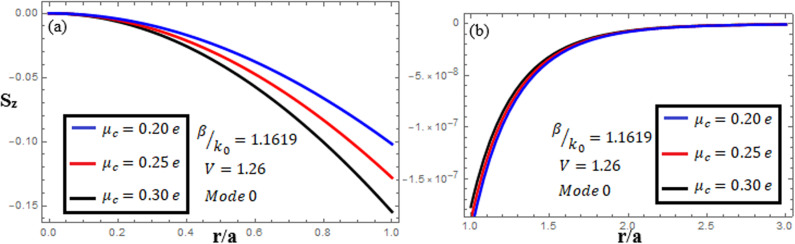
Energy flux “$S_{z}$”. Energy flux at different chemical potential values “$\mu_{c}=0.2e,0.25e,0.3e$” with chirality κ=1.75 for mode m=0.

**Fig 8:**
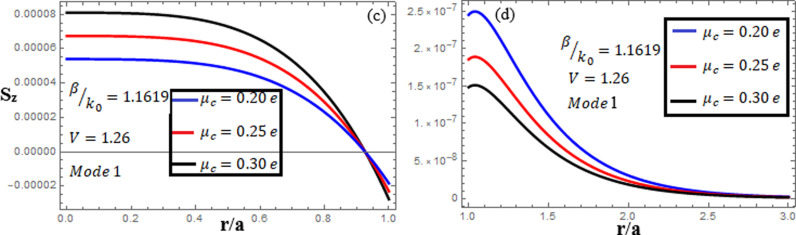
Energy flux “$S_{z}$”. Energy flux at different chemical potential values “$\mu_{c}=0.2e,0.25e,0.3e$” with chirality κ=1.75 for mode. m=1.

**Fig 9:**
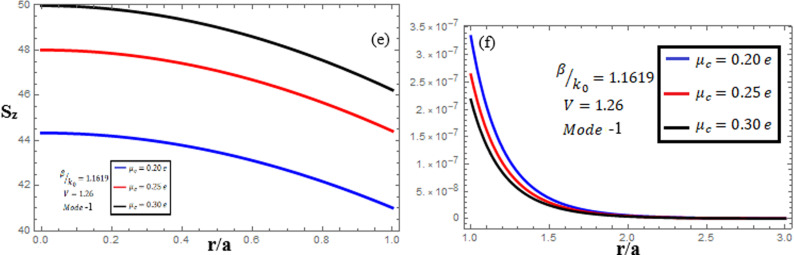
Energy flux “$S_{z}$”. Energy flux at different chemical potential values “$\mu_{c}=0.2e,0.25e,0.3e$” with chirality κ=1.75 for mode. m=−1.

Figs 7-9 examine the amplitudes of the EM field components and energy flux $S_{z}$ at normalized frequency V = 1.26, propagation constant $\beta /k_{0}=1.1619$, and chirality parameter κ=1.75 for modes 0,1,−1, respectively, at different chemical potential values: $\mu_{c}=0.2e,0.25e,0.3e$. From Fig 7, it can be seen that for mode m=0, energy flux $S_{z}$ is negative in both the core and cladding regions, and it is moving in opposite directions. From Fig 8, it can be observed that for mode m=1, energy flux $S_{z}$ has a positive value in the core away from the interface, a zero value at the center, and a negative value near the interface, and in the cladding, $S_{z}$ is positive near the interface and it decreases as it moves away from the interface. For mode m=−1, from Fig 9, it can be noted that $S_{z}$ is positive in the core region, but near the interface it decreases. In the cladding, $S_{z}$ is positive near the interface, and far from the interface, it has a zero value.

**Fig 10:**
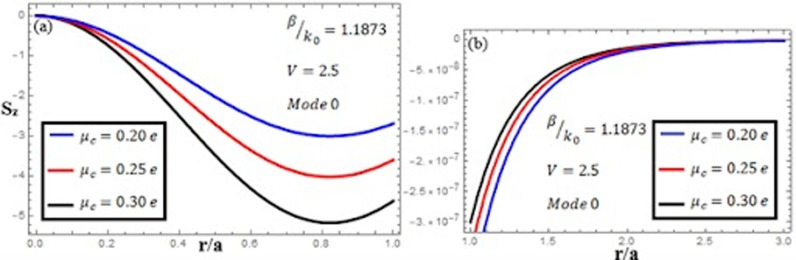
Energy flux “$S_{z}$”. Energy flux at different chemical potential values “$\mu_{c}=0.2e,0.25e,0.3e$” with chirality κ=1.75 for mode. m=0.

**Fig 11:**
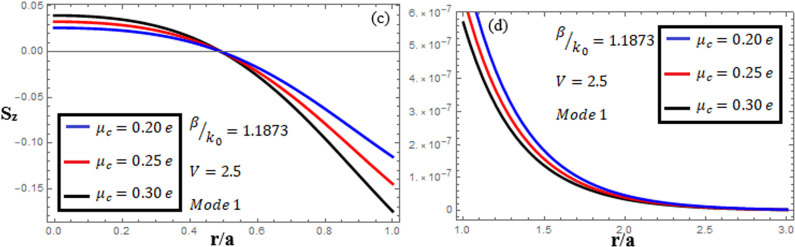
Energy flux “$S_{z}$”. Energy flux at different chemical potential values “$\mu_{c}=0.2e,0.25e,0.3e$” with chirality κ=1.75 for mode. m=1.

**Fig 12:**
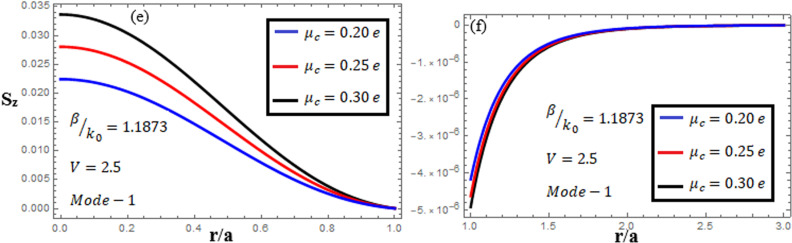
Energy flux “$S_{z}$”. Energy flux at different chemical potential values “$\mu_{c}=0.2e,0.25e,0.3e$” with chirality κ=1.75 for mode. m=−1.

Figs 10-12 examine the amplitudes of the EM field components and energy flux $S_{z}$ at normalized frequency V = 2.5, propagation constant $\beta /k_{0}=1.1873$, and chirality parameter κ=1.75 for modes 0,1,−1, respectively, at different chemical potential values: $\mu_{c}=0.2e,0.25e,0.3e$. From Fig 10, it can be seen that for mode m=0, energy flux $S_{z}$ is negative in both the core and cladding regions, and it is moving in opposite directions. From Fig 11, it can be observed that for mode m=1, energy flux $S_{z}$ has a positive value in the core away from the interface, a zero value at the center, and a negative value near the interface, and in the cladding, $S_{z}$. is positive near the interface and decreases as it moves away from the interface. For mode m=−1, from Fig 12, it can be noted that $S_{z}$ is positive in the core region away from interface, but near the interface, it decreases. In the cladding, $S_{z}$ is negative near the interface, and far from the interface, it has a zero value.

**Fig 13:**
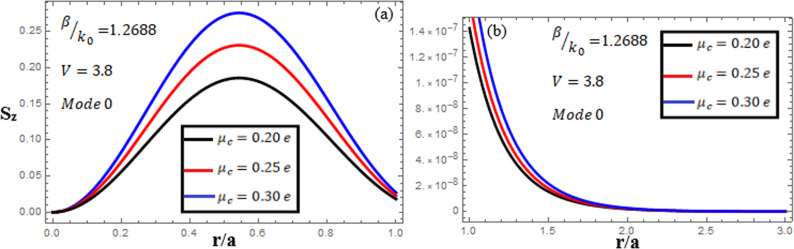
Energy flux “$S_{z}$”. Energy flux at different chemical potential values “$\mu_{c}=0.2e,0.25e,0.3e$” with chirality κ=2.0 for mode. m=0.

**Fig 14:**
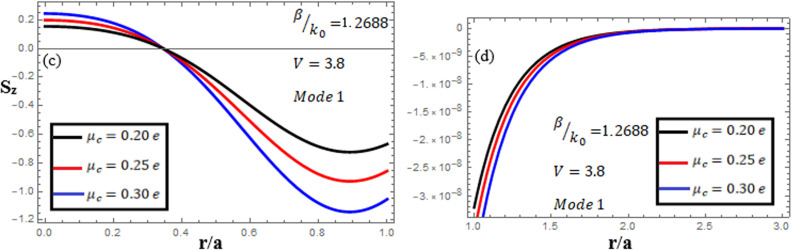
Energy flux “$S_{z}$”. Energy flux at different chemical potential values “$\mu_{c}=0.2e,0.25e,0.3e$” with chirality κ=2.0 for mode m=1.

**Fig 15:**
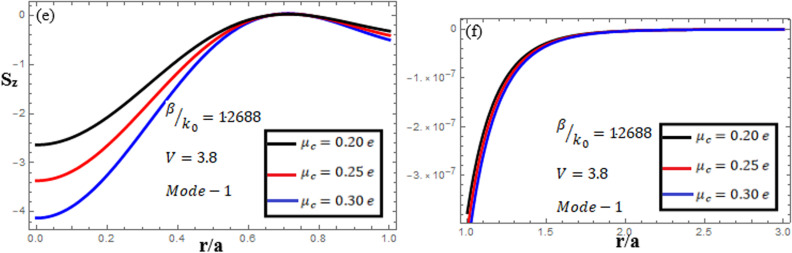
Energy flux “$S_{z}$”. Energy flux at different chemical potential values “$\mu_{c}=0.2e,0.25e,0.3e$” with chirality κ=2.0 for mode m=−1.

Figs 13-15 examine the amplitudes of the EM field components and energy flux $S_{z}$ at normalized frequency V = 3.8, propagation constant $\beta /k_{0}=1.2688$, and chirality parameter κ=2.0 for modes 0,1,−1, respectively, at different chemical potential values: $\mu_{c}=0.2e,0.25e,0.3e$. From Fig 13, it can be seen that for mode m=0, energy flux $S_{z}$ is positive in the core (first, it has a zero value at the center, and it increases to a maximum value away from the center, and then it decreases from the maximum value to a small value at the interface), and in the cladding, $S_{z}$ is positive near the interface and decreases to zero near the center. From Fig 14, it can be observed that for mode m=1, energy flux $S_{z}$ has a positive value in the core near the center, a zero value at the center, and a negative value near the interface, and in the cladding, $S_{z}$ is negative near the interface and moves toward a zero value as it moves away from the interface. For mode m=−1, from Fig 15, it can be noted that $S_{z}$ is negative in the core region away from the interface, but near the interface, it moves toward a zero value, and in the cladding, $S_{z}$ has a negative value close to the interface and it moves toward a zero value as it becomes far from the interface.

**Fig 16:**
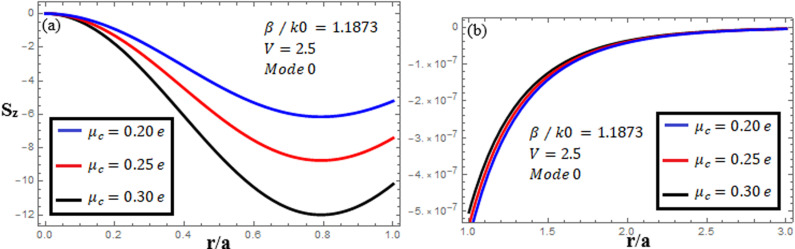
Energy flux “$S_{z}$”. Energy flux at different chemical potential values “$\mu_{c}=0.2e,0.25e,0.3e$” with chirality κ=2.0 for mode m=0.

**Fig 17:**
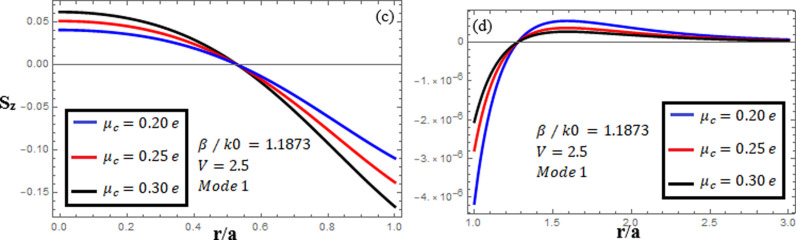
Energy flux “$S_{z}$”. Energy flux at different chemical potential values “$\mu_{c}=0.2e,0.25e,0.3e$” with chirality κ=2.0 for mode m=1.

**Fig 18:**
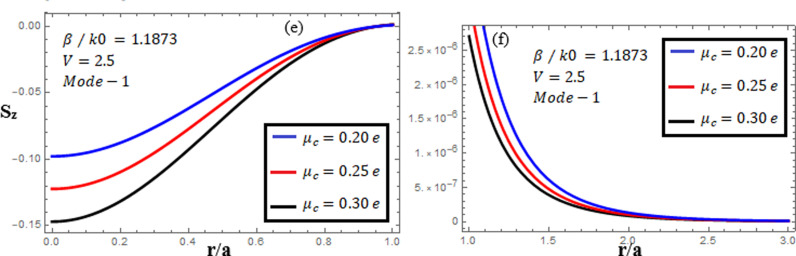
Energy flux “$S_{z}$”. Energy flux at different chemical potential values “$\mu_{c}=0.2e,0.25e,0.3e$” with chirality κ=2.0 for mode m=−1.

Figs 16-18 illuminate the amplitudes of the EM field components and energy flux $S_{z}$ at normalized frequency V = 2.5, propagation constant $\beta /k_{0}=1.1873$, and chirality parameter κ=2.0 for modes 0,1,−1, respectively, at different chemical potential values: $\mu_{c}=0.2e,0.25e,0.3e$. From Fig 16, it can be seen that for mode m=0, energy flux $S_{z}$ has a negative value in the core and cladding regions. From Fig 17, it can be observed that for mode m=1, energy flux $S_{z}$ has a positive value in the core near the center, a zero value at the center, and a negative value near the interface, and in the cladding, $S_{z}$ is negative near the interface, zero at the center, and positive far from interface. This exotic feature was first found in the air–NIM circular interface [41], and now, in our research work, it has been found in the graphene–NIM circular interface. For mode m=−1, from Fig 18, it can be noted that $S_{z}$ is negative in the core region away from the interface, but near the interface, it moves toward zero, and in the cladding, $S_{z}$ has a positive value close to the interface and a zero value far from the interface.

It should be noted that the fabrication technique of the proposed structure is same as the graphene nano-wire structure which are briefly discussed in literature [58]. For depositing thin layers of graphene on substrate chemical vapor deposition (CVD) technique is used. CVD is used for the growth of graphene on the substrate by exposing it to precursor gasses under controlled conditions [59]. After depositing the graphene thin layers on substrate Lithography or metamaterial fabrication technique is used to fabrication of chiral nihility material and define the required waveguide structure on substrate and Etching technique is helpful for the removal of unwanted materials from substrate [60]. For generation of electromagnetic waves of required frequency range signal antennas and generators can be used. Then vector network analyzers can be used to measure the waveguide characterization like scattering parameters (S-parameters). Scanning probe microscopy can be used for measurement of electromagnetic field distribution. At the end of our experiment verify the existence of forward and backward predicted modes and their propagation characteristics.

## Conclusion

The characteristics of guided modes in graphene-coated chiral nihility material-filled waveguides have been examined theoretically. The formulas and field equations related to the guided modes, graphene, and chiral nihility material have been derived in detail. The dispersion relations, dispersion curves (for both forward and backward modes), and energy flux values for guided modes have been obtained and discussed. It is found that with the addition of graphene in place of conventional dielectric material with NIM, this structure supports very slow waves with low energy losses with the strong effect of the chemical potential of graphene in wave propagation properties. The combination of chiral nihility with graphene metamaterial opens a new gate for manufacturing high-performance and ultra-compact devices which can take optoelectronics a step further forward. This integration will also be used to develop innovative applications like in metasurfaces for beam manipulation and steering, electromagnetic shielding by designing tunable reflectors and absorbers, enhancing the sensitivity of chemical sensors and biosensors, and 5G and terahertz compact antenna communications. It enables the tunability of conductivity through chemical and electrical doping. Furthermore, the conductivity of graphene can be tuned by changing its chemical potential. Interestingly, the dispersion curves of the guided modes intersect each other, so this work has potential applications in the development of novel optical fiber devices, communication systems, and chiral sensing systems at the THz frequency.
